# Vulnerable Atherosclerotic Plaque: Is There a Molecular Signature?

**DOI:** 10.3390/ijms232113638

**Published:** 2022-11-07

**Authors:** Roxana Mihaela Chiorescu, Mihaela Mocan, Andreea Ioana Inceu, Andreea Paula Buda, Dan Blendea, Sonia Irina Vlaicu

**Affiliations:** 1 Internal Medicine Department, Iuliu Hatieganu University of Medicine and Pharmacy, 400012 Cluj-Napoca, Romania; 2 Department of Internal Medicine, Emergency Clinical County Hospital, 400006 Cluj-Napoca, Romania; 3 Department of Pharmacology, Toxicology and Clinical Pharmacology, Iuliu Hatieganu University of Medicine, 400349 Cluj-Napoca, Romania; 4 Department of Cardiology, Nicolae Stăncioiu Heart Institute, 400001 Cluj-Napoca, Romania; 5 Department of Cardiology, Iuliu Hațieganu University of Medicine and Pharmacy, 400437 Cluj-Napoca, Romania

**Keywords:** atherosclerosis, vulnerable plaque, inflammatory biomarkers, proteomics, micro-RNAs, oxidative stress

## Abstract

Atherosclerosis and its clinical manifestations, coronary and cerebral artery diseases, are the most common cause of death worldwide. The main pathophysiological mechanism for these complications is the rupture of vulnerable atherosclerotic plaques and subsequent thrombosis. Pathological studies of the vulnerable lesions showed that more frequently, plaques rich in lipids and with a high level of inflammation, responsible for mild or moderate stenosis, are more prone to rupture, leading to acute events. Identifying the vulnerable plaques helps to stratify patients at risk of developing acute vascular events. Traditional imaging methods based on plaque appearance and size are not reliable in prediction the risk of rupture. Intravascular imaging is a novel technique able to identify vulnerable lesions, but it is invasive and an operator-dependent technique. This review aims to summarize the current data from literature regarding the main biomarkers involved in the attempt to diagnose vulnerable atherosclerotic lesions. These biomarkers could be the base for risk stratification and development of the new therapeutic drugs in the treatment of patients with vulnerable atherosclerotic plaques.

## 1. Introduction

Atherosclerosis and its clinical manifestations, coronary and cerebral artery diseases, are the most common cause of death in developed countries. Atherosclerosis is an inflammatory process resulting in subintimal lipid accumulation that may cause lumen stenosis. The plaque rupture and subsequent thrombosis results in acute complications, such as myocardial infarction (MI) or a stroke. An atherosclerotic plaque with an increased risk for rupture and thrombosis is called a vulnerable plaque [1]. 

When studying the association between the appearance of the atherosclerotic plaques and their risk of rupture, it was found that minor plaques, but rich in lipids, are more likely to become unstable due to the inflammatory reaction maintained by the interaction between lipoproteins, monocytes, macrophages, T lymphocytes, and vascular wall cells. Therefore, traditional imaging techniques that characterize the plaque by its appearance and size are not enough to predict the risk of rupture and the development of an acute thrombotic event. Thus, it is necessary to identify novel biomarkers and imaging methods, such as intravascular imaging, that are correlated with the instability of the atheroma plaques [2,3,4].

Therefore, the risk stratification for patients susceptible to an acute vascular event is very important in therapy management and it becomes necessary to use a multimarker strategy based on biomarkers involved in different pathways of atherosclerosis. 

This review will emphasize the most recent progressions in understanding the atherosclerosis process, regarding the molecular signature of the different pathophysiological stages. The combined use of different serum and tissue biomarkers might be the key to identifying vulnerable atherosclerotic plaques and to obtain an accurate patients’ stratification for the risk of acute vascular events. 

## 2. Pathophysiological Mechanisms and Molecular Events during the Development of Vulnerable Atherosclerotic Plaques: Plaque Erosion versus Plaque Fissure

The endothelial lining of the vascular tree is cardinal in preserving homeostasis in the cardiovascular system, since it governs flow-mediated vasodilation, vascular permeability, and recruitment of immune cells in the subendothelial space [5]. Healthy vascular endothelial cells (EC) exposed to uniform laminar shear stress, found in a basal antiinflammatory quiescent non-proliferative state, have been noted to display an atheroprotective phenotype [6]. Contrastingly, exposure to enzymatically modified LDL, oxidative stress, advanced glycation end products, and disturbed shear stress will activate EC. Endothelial dysfunction implies EC with an NF-κB signaling driven atheroprone phenotype: Accelerated cellular turnover, enhanced cell surface expression of adhesion molecules (VCAM-1, ICAM-1, and P-selectin), and production of proinflammatory receptors (toll-like receptor 2—TLR2), chemokines (MCP-1 and IL-8), and potent prothrombotic molecules (tissue factor and plasminogen activator inhibitor 1) [7,8,9].

In atherosclerosis, susceptible regions are characterized by compromised endothelial barrier integrity—deriving from diminished expression of endothelial nitric oxide synthase and superoxide dismutase SOD [6]. Thereupon, fatty streak lesion formation, the earliest event in the pathogenic sequence of atherogenesis, consists of the focal permeation and trapping of circulating modified (via oxidation, glycation, enzymatic) lipoprotein particles in the subendothelial space. Circulating monocytes are recruited into the intima, where they will withstand differentiation into macrophages—the source for later transformation into lipid-laden foam cells [7,9].

Phenotype M1 macrophages are proinflammatory and their presence is correlated with vulnerable plaques, whereas M2 macrophages are rather antiinflammatory and preclude foam cell formation. 

Recent data from Edsfeldt et al. showed that in vulnerable carotid plaques transcription factor interferon regulatory factor-5 drives CD11c+ proinflammatory macrophage activation, subsequent production of chemokines CCL2 and CCL4, and concomitant inhibition of the efficient efferocytosis apparatus [10]. Previously, the same group had reported the association between CD163+ macrophages and vulnerable plaque phenotype in a large cohort of 200 human carotid plaques [11].

Oxidized or enzymatically modified LDL prompts not only the participation of the innate immune system through scavenger-receptor internalization of the modified lipid particles, signal pattern recognition receptors (PRRs), or mast cells, but also the contribution of the connectors between the innate and the adaptive immune systems—the dendritic cells. In addition, trapping of circulating modified LDL particles in the subendothelial space abets the involvement of adaptive immunity: subsets of T lymphocytes (proinflammatory THELPER1 and antiinflammatory TREGULATORY cells) and B lymphocytes (atheroprotective B-1 cells and proatherogenic B-2 cells) [12,13]. 

In symptomatic human atherosclerotic carotid plaques, T cells were found to be more prominent, more activated, differentiated, and exhausted (expressing high levels of PD-1—marker of T cell exhaustion) compared to their blood counterpart [9].

A more complex lesion—the fibromuscular plaque—arises after chemokines secreted by ECs and macrophages incite the migration of smooth muscle cells (SMCs) from the media into the intima, where they will proliferate and synthesize extracellular matrix (ECM) macromolecules (interstitial collagen, elastin, proteoglycans, and glycosaminoglycans) or, in other words, the fibrous cap [9]. A defective efferocytosis process elicits the formation of the necrotic, lipid-rich core in parallel. Mature atherosclerotic plaques can either be stable lesions (featuring a thick fibrous cap and less lipid and inflammatory cell content) or display structural plaque instability, through the proteolytic modification of its ECM components. The evolution of vulnerable plaques potentially culminates in thrombus formation due to frank plaque rupture or due to superficial plaque erosion [14]. Culprit lesions of fatal thrombi in coronary arteries display decreased VSMC synthesis and increased breakdown (by enhanced levels of collagen-degrading enzymes overexpressed by macrophages) of the collagen fibrils that would have shielded the plaque from rupture [15]. However, uncommonly, acute coronary syndromes (ACS) can also occur without apparent thrombus [16]. 

The vulnerability index, a calculated histological ratio, was proven an efficient tool to discern between vulnerable and stable atherosclerotic lesions; this index measures the existing elements decisive for plaque rupture: plaque destabilizers (macrophages, hemorrhage, and lipids) and stabilizers (smooth muscle cell and collagen) [17].

An even more complex classification of vulnerable plaques discriminates between disrupted fibrous cap lesions, represented by (i) plaque ruptures and (ii) calcified nodules, and intact fibrous cap lesions (plaque erosions) [16].

The pathogenesis of superficial plaque erosion and that of ruptured plaque (also known as thin-capped fibroatheroma (TCFA)) are dominated by distinct molecular events and cellular participants. TCFAs possess large lipid cores covered by a thin fibrous cap (under 60 microns) and a fibrin-rich thrombus [14,18], which are usually infiltrated by inflammatory cells (mostly macrophages) provoking proteolytic activity, and hence, ECM degradation [19]. VSMC senescence and apoptosis is seen in the “shoulder” regions of such atherosclerotic plaques, with VSMC senescence being steered by decreased expression of telomeric repeat-binding factor-2 (TRF2), followed by telomere dysfunction and DNA damage [20].

In contrast to TCFAs, in lesions with superficial erosion there is a scarcity of macrophages, a substantial supply of dedifferentiated VSMCs and ECM (hyaluronan, versican, type III collagen), little or no lipid core and no disruption of the fibrous cap [21]. In the superficial plaque erosion, the center-stage belongs to endothelial cells and to granulocytes, according to in vitro data provided by Quillard and colab.; they hypothesized a “two hit” molecular sequence in the superficial plaque erosion, the first consisting of TLR2 mediated EC injury, EC detachment, or apoptosis [8]. The second hit is represented by the recruitment and adherence of granulocytes to the intimal injured site and subsequent release of reactive oxygen species, DNA, histones or—in other words—the formation of neutrophil extracellular traps (NETs). NETs are able to forge a nidus, amplifying thrombosis and expanding the local inflammatory response [8,18]. 

Most patients with plaque erosion will have a non-ST-segment elevation ACS as clinical presentation, while ST-segment elevation myocardial infarction (STEMI) will occur in most of the individuals with coronary ruptured TFCAs [13]. Plaque erosion was also associated more with women than with men. The formidable efficacy of lipid lowering therapy in the last decade has led to a shift in the clinical presentation of ACS: From a preponderance of STEMI to an ascendency of NSTEMI. Harmoniously, a synchronicity in the mechanistic phenomenon was also observed: The rise of plaque erosion cases (which now account for a third of ACS—namely the residual burden despite a quality lipid control) in parallel with the regression of TCFA ruptures [18]. 

Microcalcifications in the atherosclerotic plaques begin with matrix vesicles and apoptotic bodies released during the death of macrophages and proliferative phenotype SMCs [22,23]. Microcalcifications then fuse into sizable masses, expanding from the deeper region of the necrotic core into the surrounding ECM, and ultimately assemble into calcified sheets or plates. Patients with larger calcifications are oftentimes asymptomatic [22,23]. The extent of calcification is inversely correlated to macrophage infiltration. Vulnerable plaques prone to complicate rupture or intraplaque hemorrhage usually display either multiple and superficial calcifications (small spherical calcification or arc-shaped calcification) or calcified nodules [23,24].

## 3. Imaging Biomarkers of Vulnerable Plaques

Imaging atherosclerosis has evolved along with morphological findings in vulnerable plaques. There are at least four anatomopathological features strongly associated with the risk of rupture and worth targeting by different imaging methods. These risk features include fibrous cap micro-calcifications, cholesterol crystals, apoptosis of intraplaque macrophages, and endothelial shear stress distribution [25]. Imagery of vulnerable plaque comprises anatomical imaging and molecular imaging. Anatomical imaging is classified as invasive and non-invasive. 

Catheter-based contrast angiography (CCA) is the gold standard for the coronary vessels’ invasive evaluation, having the advantage of high spatial and temporal resolution. Currently, fractional flow reserve (FFR) is the most powerful tool in assessing the potential ischemic risk of lesions and in reducing future clinical events in patients with symptoms [25]. This method uses the intracoronary pressure to establish whether or not the flow is limited by the presence of a plaque and diameter reduction. It represents the difference between the pressure upstream and downstream of the lesion after administration of a vasodilator to augment flow [14,26]. Unfortunately, FFR is not a good predictor of acute events. Studies have shown that the vulnerability of the plaque is strongly determined by its propensity to rupture and with individual systemic characteristics, such as inflammation [26]. Thus, other supplementary intravascular imaging techniques, called catheter-based imaging techniques were develop to further characterize the plaque and its surroundings [27]. Optical coherence tomography (OCT) provides detailed images of the fibrous cap using near infrared light delivered via a fiber optic wire. The images provided by OCT correlate well with histological features high signals in the fibrous cap signify macrophage adherence and thrombus formation [28,29]. Near infrared spectroscopy (NIRS) is another catheter-based invasive technique. NIRS does not require a blood free field and uses wave scatter to produce a gradient map corresponding to the probability of adjacent lipid. The resultant lipid-core burden index describes the ratio of high lipid content in adjacent structures against the total study area [30]. Modern probes are combined with intravascular ultrasound (IVUS) to provide structural context to the morphological data. IVUS is a more established and, consequently, cost-effective technique than OCT and NIRS [31]. Calcification could be easily detected by IVUS combined with virtual histology or MicroPure IVUS which is rather useful for carotid plaques [32].

Non-invasive classical imaging methods reliably assess atherosclerotic plaques in clinical practice with little additional effort. Coronary computed tomography angiography (CCTA) or cardiac magnetic resonance imaging (CMR) are safe and offer a reliable alternative to catheter angiography [27]. These imaging modalities can simultaneously image the vessel wall and any surrounding atherosclerosis. Similarly, ultrasound (US) can identify the plaque location and characteristic and is a reliable method to quantify the degree of stenosis, rather useful to assess peripheral arteries than coronary territory. 

Characteristics of vulnerable plaques as identified by CCTA are synthesized as follows: low attenuation plaque (<30 Hounsfield units), napkin-ring sign (NRS), spotty calcium, and positive remodeling [33]. Low-attenuation plaque is associated with a necrotic core rich in lipids, because of necrosis and apoptosis of macrophage foam cells [33]. A thin fibrotic cap above a necrotic core is often described as an NRC on CCTA, manifesting as a low-attenuation area surrounded by a high-attenuation rim, which are characteristics of high-risk plaques [34]. Finally, spotty calcification identifies inflamed areas of confluent coronary calcification and microcalcification and are the consequence of an inflammatory microenvironment [34]. MR spectroscopy (MRS) combines the spatial imaging obtained from MRI with spectral analysis to detect the chemical composition and metabolic state of cardiovascular tissue. MRS is able to detect a range of atoms, including 1-Hydrogen (1H), 31-Phosphorus (31P), and 13-Carbon (13C) and identifies holesteryl esters as the major class of lipids found in the lipid-rich necrotic core of vulnerable [35]. Non-invasive imaging is useful for the clinical decision making, risk stratification and establish the opportunity for intervention by a multidisciplinary team [27].

Morphological imaging characteristics of vulnerable plaques are the progression of plaque volume over time, and its morphology with irregular surface and the presence of calcifications, ulcerations, and intraplaque hemorrhage [31]. Non-invasive imaging methods of vulnerable plaques classified by the targeted mechanism of vulnerability are mentioned in Table 1.

For optimizing the risk stratification, new molecular imaging methods to assess the vulnerable plaques were developed. Functional-molecular evidence, acquired through the performance of PET techniques, could contribute significantly to the assessment of several crucial mechanisms of plaque vulnerability, such as inflammatory cell infiltration, neo-angiogenesis, hypoxia, apoptosis, and calcifying activity [42]. Htun et al. showed that in vivo monitoring of intraplaque hemorrhage is possible by using fluorescence emission computed tomography (FLECT) and detecting near infrared autofluorescence [41].In order to improve risk assessment accuracy, combined methods using artificial intelligence and imagery were developed. Radiomics represents the process of extraction of quantitative characteristics for radiological images and the conversion into datasets that could be exploited using data extraction and deep learning algorithms which integrate clinical and genetical information from a multidimensional database, useful not only for statistical analysis of general population, but also for individual risk assessment for each and every patient [36]. Radiomic features can be calculated using both the original images as well as mathematical transformations of the original data, such as wavelet decompositions. Wavelet transformation decomposes the data into high- and low-frequency components, which describe the pattern and rate at which attenuation changes along spatial directions [43]. 

Unfortunately, imagistic biomarkers do not fulfill ideal the ideal biomarker features, having drawbacks such as their invasive character, interpretation being visually performed, being subjective, being influenced by experience, high costs, and low availability [44].

## 4. Circulating Biomarkers of Vulnerable Plaques

### 4.1. Systemic Inflammatory Biomarkers

**Figure 1:**
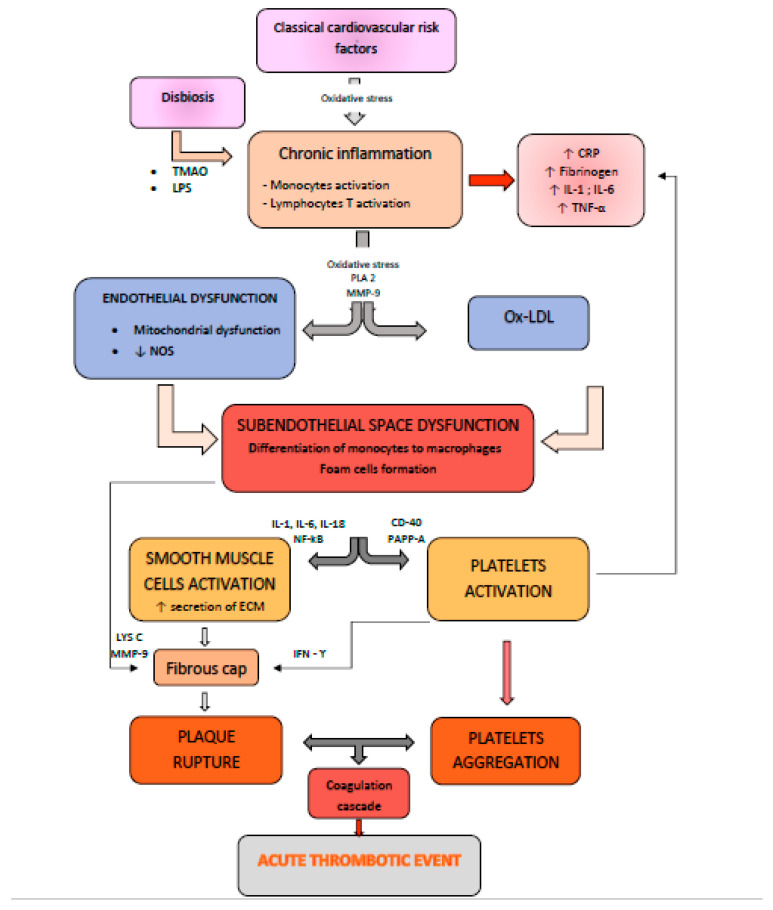
Circulating biomarkers of the vulnerable plaque and the main mechanisms involved in atherosclerotic plaque development. CRP-C, reactive protein; IFN-γ, Interferon-γ; IL-1, Interleukin-1; IL-18, Interleukin-18; IL-6, Interleukin-6; LPS, lipopolysaccharide; LYS-C, lysozyme c; MMP-9, matrix metalloproteinase-9; NOS, nitric oxide species; PAPP-A, pregnancy associated plasma protein A; PLA-2, Phospholipase A2; TMAO, Trimethylamine-N-oxide; TNF-α, Tumor necrosis factor alpha.

Inflammation plays a crucial role in the development of vulnerable atherosclerotic plaques (Figure 1). The main inflammatory biomarkers and their role in atherosclerosis, with their inherent clinical implication in the diagnosis and stratification of patients at risk for cardiovascular events, are presented in Table 2.

#### 4.1.1. C-reactive Protein 

C-reactive protein (CRP) is an important inflammatory modulator that is activated in acute inflammation and contributes to host defense. In the circulation, CRP is mainly found as the native pentameric form (pCRP), whereas in the tissues it dissociates into non-soluble monomers (mCRP). CRP was associated with atherogenesis and atherothrombosis, whereas pCRP and mCRP exert different functions in atherosclerosis progression [45]. 

CRP was proved to be a contributor to endothelial cell activation and dysfunction. Locally, CRP is produced by the cells nearby the atherosclerotic plaque, like the SMCs, the macrophages, and the ECs. pCRP activates proinflammatory pathways, mainly stimulation of NF-κB pathway, that lead to the expression of VCAM-1, ICAM-1, E-selectin, and monocyte chemotactic protein-1 (MCP-1) [45]. In vitro studies showed that CRP decreases endothelial nitric oxide synthase (eNOS) activity and secretion, inhibits prostacyclin production, and favors the uptake of oxidized [46,47,48]. mCRP favors endotelin-1 production, thus altering endothelial-dependent vascular relaxation [45]. 

Additional studies demonstrated that CRP has a prothrombotic activity, and thus, is an important determinant for acute cardiovascular events. CRP increases the expression of tissue factor (TF) in monocytes, SMCs and ECs, a thrombosis initiator after vascular injury. CRP alters the fibrinolytic activity, upregulates the activity of plasminogen activator inhibitor-1 (PAI-1), and decreases tissue plasminogen activator (tPA) expression. Other prothrombotic mechanisms activated by the CRP are the downregulation of NO production and bio-availability, modulation of prostanoid balance and activity, and the enhancement of reactive oxygen-species (ROS) production [46]. mCRP is the predominant form involved in atherothrombosis by inducing platelet activation and adhesion and thrombus growth, whereas pCRP has not been associated with thrombogenesis [45]. CRP is a ubiquitarian biomarker of inflammation tightly linked to IL-6 and may be useful for risk stratification in patients with metabolic syndrome, as previously showed by our group [49,50]. 

In patients presenting with acute myocardial infarction mCRP levels were increased, suggesting that mCRP could become a biomarker for the diagnosis and prognosis of myocardial infarction [51]. 

#### 4.1.2. Fibrinogen

Fibrinogen is a glycoprotein consisting of three different polypeptides, involved in coagulation cascade. Also known as coagulation factor I, it is secreted in a constitutive pattern in the liver, but the plasmatic level increases in inflammatory conditions; thus, fibrinogen is also an acute phase protein. Epidemiological studies indicated that raised fibrinogen concentration is a risk factor for the development of atherosclerosis and cardiovascular morbidity and mortality. The main proatherogenic effects of fibrinogen are: development of a proinflammatory environment in the endothelium, endothelial dysfunction, platelet activation, overexpression of adhesion molecules on vascular endothelium, enhancement of macrophage and LDL cholesterol infiltration in the atherosclerotic plaque, migration and proliferation of SMCs, and stimulation of angiogenesis [52]. 

In stable coronary artery disease, higher fibrinogen concentration was associated with the presence, severity, and complexity of coronary lesions, as evaluated by the Syntax Score [53]. In acute coronary syndromes, fibrinogen level at admission was independently associated with death risk [54]. Elevated fibrinogen plasmatic levels in patients with coronary artery disease undergoing percutaneous coronary intervention were predictive of long-term all-cause and cardiac mortality, especially in patients presenting with diabetes mellitus (DM) and pre-DM [55]. 

#### 4.1.3. Cytokines

Cytokines are proteins with a low molecular weight involved in inflammation pathways that include the interferons (IFN), interleukins (IL), chemokines, and tumor necrosis factors (TNF) [56]. Regarding atherosclerosis development, cytokines are responsible for immune cell recruitment, atherosclerotic plaque progression and stability, based on their proatherogenic or antiatherogenic effects. Proinflammatory cytokines that are associated with atherosclerotic disease progression are IL-1α, IL-β, IL-6, IL-18, TNF- α, and IFN-γ. Antiinflammatory cytokines that have shown cardioprotective effects are IL-10, IL-13, IL-33, and TGF-β [56]. The progression to an atherosclerotic vulnerable plaque, traditionally characterized by a thin cap fibroatheroma, a large necrotic core and micro-vessels development within the plaque, is governed by the balance between these proinflammatory and antiinflammatory molecules [57]. 

##### TNF-α

TNF-α is a proatherogenic cytokine secreted by macrophages, lymphocytes, and vascular SMCs. The proatherogenic effects involve the overexpression of adhesion molecules, i.e., ICAM-1, VCAM-1, and MCP-1, in the vascular wall and the activation of the scavenger receptor class A (SRA), increasing the uptake of the oxidized LDL in macrophages [58]. Moreover, in the arterial wall, the activation of the TNF receptor 1 (TNFR) favors the atherosclerotic process both in the early-phase and late-phase, through the augmentation of adhesion molecule and chemokine expression and the promotion of SMC migration and proliferation [59]. Kaptoge S. et al. concluded that TNF-α is associated with an increased risk of coronary heart disease independent of conventional risk factors [60]. 

##### IFN-γ

IFN-γ, produced by T cells and macrophages, activates most commonly the Janus kinase (JAK)/signal transducer and activator of transcription (STAT) pathway. It is involved in all stages of atherosclerosis development: EC dysfunction, foam cell production, oxidative stress activation, SMC proliferation, migration, and proatherogenic phenotype via platelet-derived growth factor (PDGF) overexpression and plaque destabilization [61]. Th1 cells stimulate macrophages to produce cytokines and vasoactive molecules via IFN-γ in atherosclerotic lesions. IFN-γ induces the release of matrix metalloproteases (MMPs) in macrophages and vascular cells and promotes SMC and macrophages apoptosis, resulting in weakened lesions that are prone to rupture or erosion [62]. 

##### IL-1 Family

The IL-1 family of cytokines includes proinflammatory cytokines (IL-1α, IL-1β, IL-18, IL-33, IL-36α, IL-36β, and IL-36γ), an antiinflammatory cytokine (IL-37), and three receptor antagonists (IL-1Ra, IL-36Ra, and IL-38). These cytokines exert their function by binding to the IL-1 receptor (IL-1R) family [63]. 

IL-1 is a cytokine that contributes to atherogenesis and atherosclerosis progression. The main mechanisms are: The production of VCAM-1, ICAM-1, and MCP-1, which promote monocyte recruitment in the atheroma plaque, proliferation of SMCs, local production of IL-6 and prostaglandins, vascular cell production of MMPs, and release of tissue factor with subsequently platelet adhesion and further activation of coagulation cascade [64,65]. IL-1 has two isoforms, i.e., IL-1α and IL-1β, both of which can be found in the endothelial cells and smooth muscle cells from the vascular wall, with different proinflammatory functions. Vromman A et al. demonstrated that the two isoforms of the IL-1 have distinct roles in the atherosclerosis: IL-1α is involved in the artery remodeling during the early phase of atherogenesis and IL-1β is responsible for the inflammation during atherogenesis and the development of the advanced plaque [66]. In STEMI patients, IL-1β levels were increased during the acute phase, but after PCI, they decreased rapidly and then increased slowly, reaching levels comparable to baseline after 2 months. One year after reperfusion, IL-1β was associated with impaired myocardial function and non-infarct left ventricular mass, suggesting that IL-1β could be involved in myocardial adverse remodeling and ventricular dysfunction after myocardial infarction [67]. Moreover, soluble (s) IL-1 receptors (R)2 (sIL-1R2) levels were associated with CRP serum concentration, myocardial infarct size, changes in end-diastolic and end-systolic volumes of the left ventricle, and the decrease in the ejection fraction (EF) after 4 months following STEMI [68]. 

Interleukin 18 (IL-18) is a proinflammatory cytokine that triggers IFN γ secretion in T lymphocytes and natural killer cells. In an experimental animal study, the authors demonstrated that in Apo E-/- mice, IL-18 administration leads to the development, progression and destabilization of atherosclerotic lesions involving the initiation of an inflammatory response by binding to IL-18Rα via NF-κB, the alterations in lipid metabolism, and the increase of mARN expression of the CD36, MMP-9, and NF-κB [69]. In STEMI patients undergoing PCI, the IL-18 serum levels were higher comparative with control subjects and were positively correlated with the level of peak troponin and hs-CRP. Higher IL-18 levels at admission predicted the risk of composite major adverse clinical events (MACE) at 60 days during follow-up, but not during long-term follow-up [70]. 

NOD-like receptor (NLR) family and the pyridine-containing domain 3 (NLRP3) inflammasome is a molecular complex that consists in a sensor (NLRP3), an adaptor (apoptosis-associated speck-like protein, which contains a caspase recruitment domain—ASC), and an effector (caspase-1). The effector, caspase-1, controls the cleavage of proinflammatory cytokines, such as pro-IL-1β and -18, and of gasdermin D (GSDMD). NLRP3 inflammasome activation involves the recognition of pathogen-associated molecular patterns (PAMPs) and damage-associated molecular patterns (DAMPs) by the pattern recognition receptors (PRRs), such as Toll-like receptors (TLR) and cytokine receptors (TNFR1 and TNFR2), the activation of NF-κB, and subsequently, the transcription of NLRP3, pro-IL-1β, and pro-IL-18 [71]. Other activators of NLRP3 inflammasome generate reactive oxygen species (ROS) and mitochondrial dysfunction-derived ROS (mtROS) or DNA (mtDNA) and induce the assembly and activation of NLRPAdditional mechanisms involved in NLRP3 activation are: lysosomal leakage, trans-Golgi disintegration, autophagy, and non-canonical stimuli, such as LPS or Gram-negative bacteria [72]. NLRP3 inflammasome favors vascular ECs injury, the infiltration of monocytes/macrophages, and the formation of foam cells, thus, represent an important proinflammatory contributor to atherosclerosis development and progression. In the context of atherosclerosis progression, NLRP3 inflammasome is triggered by the oscillatory shear forces that act on the endothelium, the cholesterol crystals, oxLDL endocytosis through CD36, extracellular ATP, or uric acid crystals [73]. The debris collected from ‘puff-chandelier’ lesions extruded from spontaneously ruptured atherosclerotic plaques (SRAPs) demonstrated the presence of large cholesterol crystals activated leucocytes and proinflammatory cytokines, such as NLRP3, IL-1β, IL-6, and CD68.

The cytoplasm of macrophages contained cholesterol crystals, NLRP3, IL-1β, IL-18, and caspase-This evidence suggests that the crystals of cholesterol induce an innate immune response that may be involved in plaque rupture and that NLRP3 complex is related to atherosclerotic plaque vulnerability [74]. 

In acute myocardial infarction followed by reperfusion, NLRP3 inflammasome activation was involved in ischemia-reperfusion injury due to excessive ROS production and mitochondrial dysfunction and a dysfunctional autophagic and mitophagic flux [75]. 

##### IL-6 Family

The interleukin-6 family consists in IL-6, IL-11, IL-30, IL-31, and non-IL molecules, including oncostatin M (OSM), leukemia inhibitory factor (LIF), ciliary neurotrophic factor (CNTF), cardiotrophin 1 (CT-1), and cardiotrophin-like cytokine (CLC), all of them sharing the structure and the common receptor subunit glycoprotein 130 (gp130). IL-6 has both pro and antiinflammatory properties, based on the different receptor activation, and it is secreted by lymphocytes, monocytes, adipocytes, hematopoietic cells, and endothelial cells. IL-6 pathway involves JAK/STAT activation and has important roles in atherosclerosis development and progression. IL-6 is involved in EC dysfunction, uptake of LDL and foam cells development, SMCs proliferation and migration, expression of adhesion molecules in the vascular wall, and recruitment of monocytes/macrophages. IL-6 activation leads to chronic and vascular inflammation, resulting in plaque destabilization [76]. In case of an arterial injury, local T cells secrete many proinflammatory biomarkers that may cause atherosclerotic plaque rupture. IFN increases TNF-α and IL-1 production by macrophages, thus activating inflammatory cascades. IL-6 stimulates acute-phase proteins synthesis, such as C-reactive protein, serum amyloid A, and fibrinogen, shifting local inflammation to systemic reactions [77]. 

Long-term average IL-6 has proven to be a predictor of progression of carotid atherosclerosis, independent of conventional vascular risk factors, suggesting that IL-6 could be a marker and a therapeutic target for accelerated atherosclerosis [78]. In STEMI patients treated with primary PCI, IL-6 levels were increased and soluble IL-6 receptor (sIL-6R) and sgp130 levels were decreased during the first two weeks. Higher IL-6 and a lower sIL-6R/IL-6 ratio at 24 h were indicative for larger infarct size and decreased cardiac function at 4 months [79]. 

##### NF-KB

Cytokine production and release are activated during inflammatory conditions depending on the tissue type and the molecular pathway involved. Intracellular signaling during inflammatory states comprises multiple molecular mechanisms that drive cytokine production. NF-κB is a common transcription factor that is associated with the release of different cytokines, depending on the specific inflammatory environment. Activators of NF-κB include stress, proinflammatory cytokines, microorganisms’ antigens, oxidized LDL, or free radicals. The activated NF-κB favor the transcription of cytokines, chemokines, adhesion molecules, MMPs, and acute phase proteins. In the myocardial tissue, NF-κB is activated in response to PAMPs or DAMPs released from the injured tissues that stimulate specific membrane PRRs, initiating a signaling cascade along the activator protein 1, IFN regulatory factors, and the inflammasome [80]. NF-κB signaling is involved in multiple phases of atherosclerosis, including foam cell formation, lipid metabolism disturbances, vascular SMC proliferation, vascular inflammation, vascular cell apoptosis, calcification, and plaque destabilization. NF-κB favors atherosclerotic plaque destabilization and rupture by the upregulation of expression of genes related to MMPs: MMP-2, MMP-8, and MMP-9 [81]. 

##### CD40-CD40L

CD40 is a member of the TNF receptor superfamily that is expressed on dendritic cells, lymphocyte B cells and activated T cells, macrophages, ECs, vSMCs, platelets, and fibroblasts, and is activated by its classical ligand, CD40 ligand (CD40L). CD40L is also a member of the TNF receptor superfamily and is expressed additionally on activated platelets and activated T cells. Circulating soluble CD40L (sCD40L) is released from activated platelets and T cells and plays an important role in vascular inflammation. The complex CD40-CD40L is involved in activation of ECs, SMCs, monocytes/macrophages, antigen presenting cells, and platelets. 

Alternative CD40L signaling, particularly with integrins α5β1, αMβ2, and αIIbβ3, is responsible for monocyte, fibroblast, epithelial cell, and platelet activation, which favors the expression of adhesion molecules, the production of cytokines and MMPs, the release of platelet microparticles, the aggregation of platelets, and arterial thrombi formation, depending on the different integrin stimulated [82]. 

To establish the role of CD40 found on endothelial cells during atherogenesis, Gissler MC and colab. induced EC specific deletion of CD40 in Apo E-/- mice. CD40 deficiency decreased lipid deposition and macrophage infiltration in the atherosclerotic plaque, declined leukocyte adhesion to the vessel wall and expression of adhesion molecules, and increased SMC and stable collagen fibers content in the intima, but atherosclerotic lesion size did not change. The authors concluded that CD40 found on ECs is upregulated in the time course of atherogenesis and contributes to plaque progression and inflammatory cell migration and adhesion, suggesting that endothelial deletion of CD40 promotes a phenotype of stability in the atherosclerotic plaque [83]. In another study, CD40 deficiency in myeloid cells in mice promoted a decreased inflammatory state. Lack of expression of CD40 in the macrophages resulted in a reduced atherosclerotic plaque and necrotic core and a decreased macrophage content by shifting macrophage activation towards a more antiinflammatory profile. On the contrary, oxidized LDL stimulated CD40 expression and promoted an active immune and inflammatory response [84]. CD40 found on platelets was involved in platelet-leukocyte aggregation and the release of chemokine ligand 4, but did not affect platelet aggregation [85]. 

CD40L has divergent properties depending on the activated cell type. Mice with CD40L deficiency in T cells or CD40 deficiency in dendritic cells displayed altered Th1 polarization with reduced IFN-γ production and an atherosclerotic lesion smaller and with a more stable phenotype, containing fewer T cells, smaller necrotic core, more smooth muscle cells, and a thicker fibrous cap. In patients with carotid atherosclerosis, plasmatic and advanced carotid plaques concentration of sCD40L and sCD40 were associated with IFN-γ concentration. CD40L deficiency in platelets led to a decrease in platelet deposition and fibrinogen content in the thrombi but did not affect atherosclerotic plaque size or burden [86]. 

In subjects with prevalent stroke, sCD40 and sCD40L plasmatic levels were elevated, while in patients with a prior acute myocardial infarction sCD40 were raised. Plasmatic concentration of sCD40 was associated with arterial stiffness, intima-media thickness, and carotid plaque burden and predicted the risk of future cardiovascular events over a three-year follow-up period. Plasmatic sCD40L correlated with carotid plaque progression. In human carotid plaques, sCD40 and sCD40L were positively associated with lipid and oxLDL content and proinflammatory cytokines and chemokines and negatively with calcium content, thus suggesting a role for sCD40 and sCD40L in plaque vulnerability and extracellular matrix remodeling [87]. 

##### PAPP-A

Pregnancy-associated plasma protein-A (PAPP-A) is a member of metzincin metalloproteinase superfamily that activates insulin-like growth factor (IGF) through cleavage of IGF-binding proteins (IGFBPs). 

PAPP-A increased the secretion of MCP-1, TNF-α, and IL-6 at both transcriptional and translational levels in a dose-dependent and time-dependent manner. In macrophages, PAPP-A activated the IGF-I/PI3K/Akt signaling pathway, which drives the expression and production of these proinflammatory cytokines, suggesting that PAPP-A pathway involves proinflammatory molecules in atherosclerosis development [88]. PAPP-A favors atherosclerosis development and progression and promotes lipid accumulation, EC dysfunction, SMC proliferation and migration, and vascular inflammation and increases plaque instability along with thrombus formation [89]. 

Ox-LDL stimulated PAPP-A expression in in vitro macrophages and induced the activation of NF-κB. In Apo E -/- mice with high fat diet (HFD)-induced atherosclerosis, PAPP-A deficiency ameliorated atherosclerotic lesion formation, plaque burden and collagen content and showed antiinflammatory changes in both in vivo and in vitro studies. Additionally, in mice with HFD-induced atherosclerosis, PAPP-A expression, mainly from macrophages, was upregulated in aortic plaques [90]. In another study, PAPP-A deficiency in Apo E-/- mice reduced lipid accumulation, promoted reverse cholesterol transport from macrophages, ameliorated lipid metabolism, and decreased the secretion of MCP-1, TNF-α, IL-6, and IL-1β via the inhibition of the NF-κB pathway [91]. PAPP-A favored the expression of tissue factor on cell surface and enhanced tissue factor procoagulant activity, suggesting that PAPP-A may be part of the pathophysiology of acute vascular events as a prothrombotic biomarker [92]. 

In patients presenting with acute coronary syndrome (ACS), higher plasmatic levels of PAPP-A were associated with an increased risk of major cardiovascular events at a two-year follow-up both in subjects with type-2 diabetes mellitus (T2DM) or without. Comparative to subjects without T2DM, in patients with T2DM, PAPP-A serum concentration was higher and more predictive that hs-CRP, eGFR, or LVEF < 50% for future cardiovascular events [93]. 

##### MMP

Matrix metalloproteinases (MMPs) are a family of extracellular zinc proteases that are involved in the cleavage of protein components of the extracellular matrix (ECM), favoring tissue repair and remodeling, including vascular remodeling. The group of MMPs include many types of enzymes, based on the specific substrate: collagenases, gelatinases, stromelysins, matrilysins, and several non-classified MMPs, such as MMP-12 (metalloelastase), MMP-19, MMP-20 (enamelysin), MMP-21, MMP-23 (CA-MMP), and MMP-28 (epilysin) and transmembrane MMPs. Their activity is inhibited by the tissular inhibitors of metalloproteinases (TIMPs). MMPs are activated by numerous factors, such as inflammatory molecules, e.g., IL-1β, IL17, and NF-κB, oxidative stress, and hypoxia biomarkers. In atherosclerosis development, MMPs contribute to ECM remodeling, which leads to plaque growth, increased EC permeability, dysfunction and recruitment of inflammatory cells to the endothelium, increased vascular SMC survival and proliferation, and increased plaque vulnerability with intraplaque neovascularization and prothrombotic activity [94]. 

In patients with carotid atherosclerosis, the levels of MMP-1, MMP-3, and MMP-12 were significantly increased in patients with a phenotype of vulnerable atherosclerotic plaque and had been correlated with cardiovascular and cerebrovascular major events, suggesting that MMPs can be used for clinical diagnosis and prognosis of atherosclerotic disease [95]. In human carotid plaques, the mRNA levels of MMP-2, -7, -9, and -14 were increased and the protein levels of MMP-2 and -14 were also elevated in vulnerable lesions. VEGF, bone sialoprotein 2 (BSP), indicators of neovascularization and calcification and the protein levels of extracellular regulated kinase (ERK) and protein kinase C (PKC) were overexpressed in vulnerable plaques, pointing to the role of that MMP-2 and -14 in atherosclerotic plaque vulnerability [96]. In patients with coronary atherosclerotic plaques, the circulating osteoglycin (OGN) and the plasmatic complex between neutrophil gelatinase-associated lipocalin (NGAL) and MMP9 predicted major cardiovascular events for the one-year follow-up period after coronary angiography [97]. 

NGAL is an acute phase protein, found in the granules of human neutrophils and it is expressed by macrophages, ECs, and SMCs in human carotid endarterectomy specimens. NGAL binds to MMP-9 and protects MMP-9 from inactivation. NGAL and the complex NGAL/MMP9 were also increased in vulnerable carotid atherosclerotic plaques [98]. In patients presenting with acute myocardial infarction and major cardiovascular events (MACE) at the one-year follow-up, hs-CRP, I-CAM, and MMP-9 were significantly increased and MMP-9 was the most powerful predictor for MACE [99]. In high glucose condition, the cross-talk between macrophages and SMCs caused overexpression of MMP-1 and MMP-9, increased enzymatic activity of MMP-9, increased levels of MCP-1 and activated PKC, and NF-κB signaling involved in MMPs upregulation and diminished collagen fibers assembly, suggesting that in diabetic patients, MMPs play a significant role in atherosclerotic plaque destabilization [100]. In Apo E -/- fed an HFD where unstable coronary atherosclerotic disease was induced, MMP-7 deficiency increased the incidence of sudden death, while MMP-12 knockdown promoted survival. In mice with MMP-7 or MMP-12 deficiency, atherosclerotic burden was diminished, but the burdenwas increased in TIMP-1-deficient mice. In mice with MMP-7 deficiency, myocardial tissue contained larger areas of fibrosis. These results underline the different roles for MMPs on atherosclerosis progression and the development of myocardial infarction [101]. 

##### MPO

Myeloperoxidase (MPO) is a member of the superfamily of mammalian heme peroxidase enzymes, which is principally expressed in neutrophils. The main biological effect mediated by MPO is the catalyzation for the conversion of chloride ions to hypochlorous acid in the presence of H202 [102].

In human atherosclerotic plaques of coronary artery disease, catalytically active MPO protein and its oxidation products were detected in macrophages, nearby the lipid or necrotic core, and in the vascular endothelium. MPO and oxidized proteins are more prevalent in advanced atherosclerotic plaques, such as type IV, V, and VI lesions [102]. MPO was also associated with the development of neutrophil extracellular traps (NETs) in atherosclerotic lesions, which leads to plaque progression and destabilization and vascular thrombosis [103]. MPO-derived oxidants target lipoproteins, such as LDL, promoting foam cell production, or HDL, binding apolipoprotein A-1 (Apo-A1) and generating a dysfunctional HDL molecule with proinflammatory effects, which is associated with increased expression of adhesion molecules. Moreover, MPO is involved in plaque destabilization by activation of MMPs and inactivation of TIMPs that promote the degradation of ECM. MPO is an important cause for endothelial dysfunction via production of oxidants, particularly hypochlorous acid (HOCl), hypothiocyanous acid (HOSCN), and nitrogen dioxide radical (• NO2), depletion of nitric oxide (NO) due to NO oxidase activity of MPO and non-catalytic biological activities, or cytokine-like proinflammatory effects [102]. 

In patients presenting with STEMI treated with primary percutaneous coronary intervention, elevated MPO levels predicted the occurrence of major acute cardiovascular events and the need for coronary revascularization. MPO was a better predictor for major events than NT-proBNP or left ventricle systolic function [104]. In a mouse model of atherosclerotic plaque instability, MPO activity was increased in unstable compared with stable plaque phenotype. Deletion or pharmacological inhibition of MPO activity increased fibrous cap thickness and decreased fibrin and hemosiderin, markers of intraplaque hemorrhage, without affecting lipids or lesion and circulating inflammatory cells [105]. 

MPO-mediated oxidation alters Apo-A1 atheroprotective properties and creates a dysfunctional Apo-A1 molecule that lacks ability to promote reverse cholesterol transport, decrease lipid content and macrophage number, and mediate an antiinflammatory status of the macrophages in the atherosclerotic lesion [106]. Apo-A1 containing a 2-OH at tryptophan 72 (oxTrp72-apoA1), found in 20% of the Apo-A1 in the atherosclerotic plaque, demonstrated proinflammatory activity and impaired HDL biogenesis activity in vivo. Increased oxTrp72-apoA1 levels were correlated with an increased coronary artery disease risk and cardiovascular risk, even after adjusting for traditional CVD risk factors, i.e., apoA1, MPO, and high sensitivity C-reactive protein levels, and lipid lowering medication use [107]. 

##### MCP-1/CCL-2

MCP-1/CCL-2 (monocyte chemoattractant protein 1, also called chemokine C-C motif ligand 2) is a chemokine that attracts monocytes from the bone marrow to the proinflammatory environment. MCP-1 regulates leukocyte migration into the artery wall, in the atherosclerotic lesion [108]. In human carotid atherosclerotic lesions, MCP-1 levels were associated with markers of plaque vulnerability, such as a large lipid core, decreased collagen and smooth muscle cell content, an increased burden of macrophages, intraplaque hemorrhage, and higher levels of proinflammatory, neovascularization, and matrix turnover molecules. MCP-1 levels were also correlated with symptomatic lesions and a higher risk of periprocedural and short-term major cardiovascular events [109]. In coronary heart disease patients with high levels of CCL-2, atherosclerotic lesions displayed thin cap fibroatheromas, a fibrofatty and necrotic core, decreased fibrous content, and higher eccentric plaque and plaque rupture. CCL-2 levels were positively correlated with an increased fibrofatty and necrotic core content and negatively correlated with plaque collagen content. CCL-2 overexpression was associated with genes involved in cell adhesion and Nod-like receptor signaling pathway [110]. Treatment with 15a, an antagonist on the CCL-2 receptor (CCR2), decreased the levels of circulating CCR2+ monocytes and reduced the size and the macrophages content of the carotid and aortic atherosclerotic lesions, suggesting that CCR2 antagonism might result in atherogenesis inhibition [111]. 

##### C5b-9

The complement system is involved in the development of chronic inflammatory diseases. It can be activated by the classical pathway, lectin pathway, and coagulation system pathway. All these three pathways generate C5b-9, known as the membrane attack complex. This complex is formed by C5b, C6, C7, C8, and C9 molecules. It leads to the release of chemokines and growth factors [112]. 

C5b-9 plays and important role in the pathophysiology of atherosclerosis and its tardive complications. Controlled clinical studies have demonstrated the correlation between C5b-9 level and stability and number of carotid atherosclerotic plaques as well as degree of stenosis. In humans, the appearance of activated complement products coincides with the formation of early fatty streaks in the arterial intima. C5b-9 deposits occur on macrophages, apoptotic cells, and endothelial cells found in the atherosclerotic lesion [113].

##### RGC-32

The RGC-32 (Response gene to complement 32) protein is as an important effector of the terminal complement complex, C5b-It is expressed in numerous organs and tissues. RGC-32 is involved in atherosclerosis development via vascular smooth muscle cells differentiation, proliferation and migration, inflammatory activation, angiogenesis, and macrophages modulation [114]. Cui XB et al. proved that RGC-32, mainly expressed in endothelial cells in atherosclerotic lesions, binds to NF-κB and facilitates the expression of adhesion molecules, ICAM-1 and VCAM-Deletion of RGC-32 attenuated atherosclerotic lesions development, decreased macrophages infiltration, and inhibited TNF-α-induced endothelial adhesion molecules expression and monocyte-endothelial cells interaction [115]. Recent data from Vlaicu et al. describe that RGC-32 plays a dual role in human aortic SMC, being involved in sublytic C5b-9-induced cell cycle activation and in TGF-β-induced ECM production, while also influencing the SMC phenotypic switch [116].

##### SIRT 1

SIRT 1 is a member of Silent Information Regulator 2 (Sir2) protein family. It is considered an epigenetic regulator of human physiology. Changes in SIRT 1 expression are critical in metabolic syndrome, cardiovascular diseases, cancer and neurodegeneration. SIRT 1 deficiency in ECs, vascular SMCs, and monocytes/macrophages favors oxidative stress, inflammation, foam cell formation, and impaired autophagy in vascular wall, leading to atherosclerosis progression and further destabilization of the atherosclerotic plaque. SIRT 1 exerts atheroprotective effects via eNOS production, inhibition of NF-Κb and inflammatory pathways, reduction of oxidative stress, diminished oxLDL uptake and foam cell production, and modulation of autophagy. SIRT 1 increases TIMP3 production and promotes collagen synthesis in SMCs, preventing the rupture of atherosclerotic plaque [115]. 

**Table 2:** The main biomarkers involved in atherosclerosis developments and the most common therapeutic approaches.

Biomarker	Steps in Atherosclerosis in Which the Biomarker Is Involved	Prognosis	Therapy
Hs-CRP [45,117]	Endothelial dysfunction Alteration of cholesterol metabolism Prothrombotic activity	Associated with AMI [51]	LDL-lowering therapy, including statins ACEI/ARB BB [118,119] Antidiabetic drugs, including Metformin [120,121]
Fibrinogen [52]	Endothelial dysfunction Foam cell production Platelet activation; prothrombotic activity	Prediction of all-cause and cardiac mortality [55]	
TNF-α [58,59]	Overexpression of adhesion molecules in the vascular wall Foam cell production SMCs migration and proliferation	Associated with the risk of CAD [60]	
IFN-γ [61]	Endothelial dysfunction Foam cell production Oxidative stress activation SMCs proliferation and migration Plaque destabilization	Associated with lesions prone to rupture [62]	
NLRP3/IL-1/IL-18 [64,65,69,73]	Endothelial dysfunction Foam cell formation SMCs proliferation Lipid metabolism alterations Plaque destabilization	IL-18- prediction of MACE at 60-day follow-up [70]	
IL-6 [76]	Endothelial dysfunction SMCs proliferation and migration Foam cells development	Progression of atherosclerotic lesions [78]	
NF-κB [81]	Foam cell formation Lipid metabolism disturbances Vascular inflammation Plaque destabilization	Plaque destabilization [81]	
CD40/CD4-L [82,83,84,85,86,87]	Inflammatory cell migration and adhesion Production of proinflammatory cytokines and MMPs Destabilization of atherosclerotic plaque; prothrombotic activity	Prediction of cardiovascular events and plaque progression [87]	
PAPP-A [89]	Endothelial dysfunction Vascular inflammation SMCs proliferation and migration Destabilization of the plaque	Prediction of MACE [93]	
MMPs [94]	Endothelial dysfunction SMCs survival and proliferation Increased plaque vulnerability with intraplaque neovascularization and prothrombotic activity	Prediction of major cardiovascular and cerebrovascular events [95,97]	
MPO [102]	Endothelial dysfunction Foam cell production Proinflammatory effect	Prediction of MACE [104]	
MCP-1 [108]	Monocyte migration to the atherosclerotic lesion	Correlated with symptomatic lesions and a higher risk of MACE [109]	

AMI, acute myocardial infarction; CAD, coronary artery disease; Hs CRP, high sensitivity C-reactive protein; TNF-α, Tumor necrosis factor alpha; IFN-Υ, Interferon-gamma; NLRP3, NOD-like receptor (NLR) family and the pyridine-containing domain 3; IL-1, Interleukin-1; IL-18, Interleukin-18; IL-6, Interleukin-6; NF-κB, Nuclear factor kappa-light-chain-enhancer of activated B cells; CD-40L, CD40 ligand; PAPP-A, pregnancy associated plasma protein A; MMPs, matrix metalloproteinases; MPO, Myeloperoxidase; MCP-1, monocyte chemoattractant protein 1; MACE, major adverse cardiac events; ACEI, angiotensin-converting enzyme inhibitors; ARB, angiotensin receptor blockers; BB, beta blockers.

### 4.2. Epigenetic Biomarkers of Vulnerable Plaques

The term “epigenetics” refers to modifications that affect heritable phenotypic variations without changing the genome’s DNA sequence. The production of different non-coding RNA species and covalent alterations to the structural elements of chromatin, nitrogenous bases of DNA, and chromatin that determine the pattern of gene expression are all examples of epigenetic change [122]. Finally, they help in keeping the cell identity, which is extremely important for physiological development of the cell. In the last two decades, the field of precision medicine has witnessed a growing interest in epigenetics, and epigenetic modifications have been linked to CVDs [123]. The three basic epigenetic alterations are DNA methylation, histone post-translational modifications (PTMs), and non-coding RNAs (ncRNAs) [122]. The epigenetic biomarkers of atherosclerotic plaque instability and current state of knowledge are discussed in the subsection below.

DNA-methylation dynamically regulates gene expression and involves de novo methylation, both hypo- and hypermethylation and active and passive demethylation [124]. Inflammatory status is influenced by more than 7000 genes which display hypomethylation in symptomatic plaques [125]. For example, PLA2G7, encodes lipoprotein-associated phospholipase A2 and its hypomethylation results in increased expression upon inflammation and lesion instability [126]. Importantly, the hypomethylated level of PLA2G7 significantly decreased with plaque destabilization [126]. Higher methylation levels of genes involved in plaque progression and vulnerability such as autoimmune regulator 1 (AIRE1) and arachidonate 15-lipoxygenase (ALOX12), among others, in plaques than in non-plaque intima [127]. Calcification status is influenced by hypomethylation of antiatherosclerotic genes, such as receptor activity binding protein 1 (RAMP1) [123]. 

PTMs are also implicated in atherogenesis and plaque instability. Histone H2A, histone H2B, histone H3, and histone H4 can be modified by phosphorylation, SUMOylation, methylation, acetylation, ubiquitination, GlcNAcylation, carbonylation, and ADP-ribosylation, collectively constituting the histone code [128]. Histone acetylation generally promotes gene expression, whereas histone methylation promotes repression. Histone-lysine N-methyltransferase enzyme EZH2 is thought to be associated with inflammatory status. E2H2 has been strongly associated with progression and vulnerability of atherosclerotic plaque being involved in the transcription of proinflammatory genes [125,129]. Inflammatory mediators stimulate SMCs during the formation of atherosclerosis, and their multiplication results in plaque generation. Additionally, active SMCs create a large number of extracellular matrix components causing the lesion of the fibrous cap [130]. Enhanced histone acetylation as well as a methylation reduction in H3K9 and H3K27 in the SMCs from severe atherosclerotic lesions are correlated with plaque instability [131,132]. Moreover, methylation of H3K9 and its depletion has been associated with the inhibition of the proliferation of ECs and increased angiogenesis [133].

ncRNAs are classified as microRNAs (miRNAs), long non-coding RNAs (lncRNAs), and circular RNAs (circRNAs) consisting of covalently closed continuous loops [123]. miRNAs are a class of small non-coding RNA (20–25 nucleotides) involved in gene regulation [134]. They constitute a dense network that regulates gene expression with multiple cooperative effects on a large number of genes, making it possible to control various molecular pathways at different levels [135]. However, despite the fact that miRNA activity is thought to be crucial for normal cell development and physiology, and as a result, dysregulation of miRNA function may be to blame for various human disorders, only a small number of miRNAs’ biological functions have been elucidated so far [136]. miRNAs are considered promising blood circulating biomarkers for the prognosis, diagnosis, and risk stratification and vulnerable patients’ identification [123]. Circulating miRNAs present high stability and tissue specificity and are easily detected by sequence-specific amplification being an ideal biomarker. miRNAs were recently described as biomarkers for stable vs. vulnerable carotidal artery plaques by Carballo-Perich L et al. [123]. 

**Figure 2:**
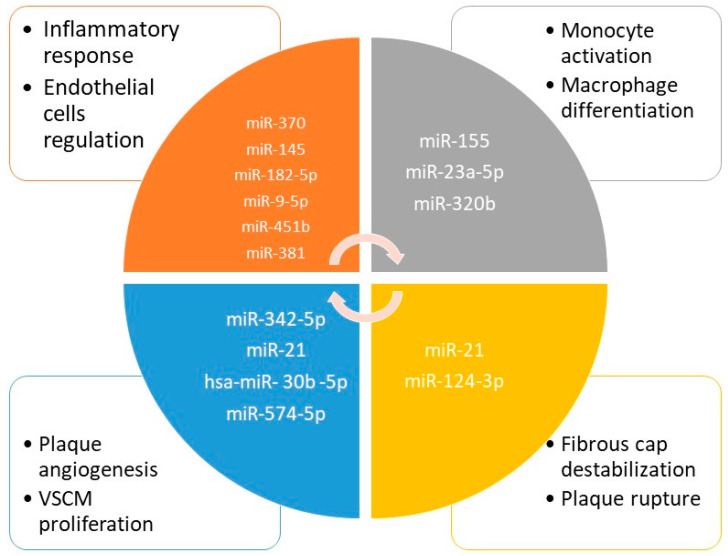
Potential role of miRNA as biomarkers of vulnerable plaque in coronary artery disease.

There is a growing interest in miRNA playing a role in coronary artery syndromes and in their potential diagnosis and prognosis role illustrated by numerous studies and reviews that covered this issue: Kumric et al. [137], Guo et al. [138], Theofilis et al. [139], and Kong et al. [140]. Some new and interesting information may be added to these descriptive reviews. Taraldsen MD et al. demonstrated that miR-15a-5p, miR-30e-5p, miR-92a-3p, miR-199a-3p, miR-221-3p, and miR-222-3p were associated with coronary plaque necrotic core volume, as identified by grayscale and radiofrequency IVUS [141]. After aerobic exercise intervention, decreased levels of miR-15a-5p, miR-93-5p, and miR-451a, and increased levels of miR-146a-5p were associated with regression of coronary plaque necrotic core. Application of a prediction tool identified that genes regulated by miR-15a-5p, miR-199a-3p, and miR-30e-5p were overrepresented in pathways related to fatty acid biosynthesis and fatty acid metabolism [141]. Moreover, He et al. showed that miR-21 was highly positively correlated with the maximum lipid core area, the number of diseased vessels, the number of macrophages, and the number of vulnerable plaques, and negatively correlated with the thickness of fiber caps [142]. Inversely, in the group presenting overexpression of miR-21, the atherosclerotic burden was higher, having more coronary artery lesions, more vulnerable plaques with necrotic lipidic core, and inflammation (macrophages) [142]. The authors showed that miR-21 was an important marker of plaque instability and might be a good predictor of acute adverse events in CAD [142]. The potential role of miRNA as biomarkers of vulnerable plaque in coronary artery disease is illustrated in Figure 2.

Recently, Singh et al. provided new data regarding the potential role of miR-122-5p and miR-223-3p as markers of plaque instability [143]. miR-122-5p and miR-223-3p were significantly upregulated both in plaque tissue evaluation and in serum, in patients with unstable coronary syndromes [143]. Some of miRNA were found to play a protective role. For example, Sun et al. showed in an experimental study on mice with acute coronary syndrome that overexpression of miR-335-5p had negative effect on macrophage by reducing the innate immune response, and thus, decreasing atherosclerotic vulnerable plaque formation. miR-335-5p targeted JAG1 through Notch signaling and promoted revascularization in mice with acute coronary disease [144]. Moreover, Yu et al. showed that miR-9 overexpression suppresses vulnerable atherosclerotic plaque and enhances vascular remodeling through negative regulation of the p38MAPK pathway via OLR1 in acute coronary syndrome [145]. These types of miRNAs could be used for targeted therapy in atherosclerosis.

The comprehensive analysis of the cell populations found in the atherosclerotic plaque, including SMCs, ECs and various types of immune cells, has had a profound influence on our knowledge of atherogenesis. Plaque instability is influenced by cell state and composition, which can also result in various clinical symptoms. Five primary plaque types were discovered by the bulk RNA-sequencing study of 654 human lesions and they are correlated with symptoms at admission [146]. This bulk data study showed that different types of plaques have various underlying cell compositions [147]. Single cell RNA sequencing (scRNA-seq) can characterize certain cell types in the atherosclerotic plaque. Innovative technologies, such as CyTOF, scRNA-seq, and CITE-Seq, allow for high-dimensional transcriptomics and proteomics with unprecedented sensitivity [148]. Experimental studies on atherosclerosis using scRNA-seq in mice have revealed a new heterogeneity of the immune and non-immune cell compartments and showed a remarkable cell adaptation and plasticity in the plaque microenvironment [147]. Kim et al. performed single-cell RNA-sequencing analysis of CD45+ leukocytes from murine atherosclerotic aorta [149]. The authors revealed that there are macrophage subpopulations with differentially expressed genes involved in various functional pathways. The paradoxical finding was that intimal non-foamy macrophages formed the major population expressing interleukin-1β leading to the conclusion that lipid-loaded plaque macrophages are not, in fact, the vulnerable inflammatory plaques [149]. Human studies performed by Fenandez et al. should that plaques from symptomatic patients were characterized by a distinct subset of CD4+ T cells and by T cells that were activated and differentiated. Moreover, some T cell subsets in these plaques presented markers of T cell exhaustion. Additionally, in plaques from asymptomatic patients, T cells and macrophages were activated and displayed evidence of IL-1β signaling [13]. Thus, the authors concluded that the identification of reparative macrophages in plaque of patients with recent CV events may contribute to the healing process following plaque disruption [13]. The benefits of using scRNA-seq for better characterizing both mice and human plaques were elegantly synthetized by some recently published systematic and descriptive reviews [147,148,150,151].

### 4.3. Gut Microbiota Biomarkers

Gut microbiota consists of all the microorganisms that live in the gastrointestinal tract, especially in the ascending colon. In adults, gut microbiota is mainly composed of five phyla: *Bacteroidetes, Firmicutes, Actinobacteria, Proteobacteria*, and *Cerrucomicrobia,* 90% of the bacterial species being from *Bacteroidetes* and *Firmicutes* species. Dysbiosis is an imbalance between microbiota composition and is related to antibiotic overuse, infections, diet and lifestyle, or specific diseases. Dysbiosis is characterized by gut mucosal increased permeability, which leads to gut bacterial DNA translocation and allow local metabolites and endotoxins to be absorbed into systemic circulation. The involvement of gut microbiota in atherosclerosis development implies bacterial metabolites, such as Trimethylamine N-oxide (TMAO), Lipopolysaccharide (LPS), and Short-Chain Fatty Acids (SCFA), that have systemic effects [152]. 

#### 4.3.1. TMAO

TMAO is generated by the enzymes produced by the gut microbiota from phosphatidylcholine, carnitine, γ-butyrobetaine, betaine, and crotonobetaine, found predominantly in animal-derived products. From the gut, formed TMA is absorbed into the portal circulation and oxidized to TMAO by the hepatic flavin containing monooxygenases FMO3 and FMO1 [152]. In the atherogenesis process, TMAO takes part in endothelial dysfunction, foam cell formation, altered lipid metabolism via inhibition of hepatic bile acid synthesis [153], and vascular inflammation via inflammatory pathways activation, including NLRP3 inflammasome [154,155]. TMAO favors atherothrombosis and promotes tissue factor expression, monocyte adhesion, and platelet hyperreactivity [156]. 

In patients presenting with acute coronary syndrome, increased concentrations of TMAO significantly predicted the risk for cardiovascular mortality during long-term follow-up. The risk for seven-year cardiovascular mortality was the highest in patients with DM and high TMAO levels at the time of presentation [157]. TMAO has been proved to be a predictor for both near and long-term risks of major cardiovascular events or mortality, suggesting it may play a role in stratifying patients presenting with suspected acute coronary syndrome [158]. Trimethyllysine (TML), a nutrient-precursor of TMAO, was also associated with major adverse cardiac events (myocardial infarction, stroke, need for revascularization, or all-cause mortality) both in the short- and long-term, independent of traditional cardiovascular risk factors [159]. In an animal study of plaque instability, TMAO levels were associated with characteristics of plaque vulnerability, such as markers of inflammation, platelet activation, and intraplaque hemorrhage. TMAO levels did not correlate with the extent of atherosclerosis or plaque composition in two animal models of atherosclerosis, suggesting that TMAO is mainly involved in proinflammatory and prothrombotic activity [160]. Higher TMAO levels were also associated with the phenotype of vulnerable atherosclerotic lesions: thinner fibrous cap thickness, higher frequency of microvessels, and increased incidence of thin-cap fibroatheroma, as assessed by optical coherence tomography (OCT). TMAO levels were significantly correlated with the incidence of thin-cap fibroatheroma, as shown by multivariate regression analysis [161]. 

#### 4.3.2. LPS

Lipopolysaccharide (LPS) is a part of the outer membrane of Gram-negative bacteria from the gut and oral cavity and contains three parts: lipid A, a core oligosaccharide, and the O-antigenic polysaccharide. It is a marker of increased gut permeability related to dysbiosis and dysfunctional gut mucosal barrier in conditions of high-fat diet, insulin resistance, atherosclerosis, and liver disease. The LPS pathway includes LPS-binding protein (LBP), CD14, and Toll-like receptor-4 (TLR4) [152]. LPS binds to upregulated TLR4 found on macrophages in atherosclerotic lesions through CD14, a lipid-binding glycophosphatidylinositol anchored membrane protein, and induces the activation of myeloid differentiation factor 88 (MyD88) signaling pathway and the release of proinflammatory cytokines and chemokines [162]. In patients with complicated >70% carotid stenosis, circulating levels of LPS and zonulin, markers of gut permeability, were significantly increased compared to controls. Antibodies against LPS, TLR4 and CD68 were positive in carotid plaque sections, and macrophages were bigger in areas positive for LPS and Toll-like receptor 4 (TLR4). LPS-incubated monocytes in a concentration similar to that found in the atherosclerotic lesion showed an increase of oxidant species and oxidized LDL production via the upregulation of NADPH oxidase 2 (Nox2), suggesting that LPS could drive atherosclerosis progression and destabilization of plaques through oxidative stress pathway [163]. Yoshida et al. demonstrated that *Bacteroides vulgatus* and *Bacteroides dorei* are significantly decreased in the gut and LPS levels are increased and negatively correlated with *Bacteroides* bacteria in patients with coronary artery disease. In atherosclerotic-prone mice, gavage with live *B. vulgatus* and *B. dorei* ameliorated atherosclerotic plaque formation and decreased gut microbial LPS production and endotoxemia, downregulating proinflammatory intestinal mechanisms [164].

#### 4.3.3. SCFA

Short-chain fatty acids (SCFAs) are produced by the gut microbiota, mainly by bacteria families, such as anaerobic *Bacteroides, Bifidobacterium, Eubacterium, Streptococcus*, and *Lactobacillus,* through fermentation of fibers and non-digestible carbohydrates. The main components of SCFAs are acetate, propionate, and butyrate. SCFA have been associated with protective effects against atherosclerosis development: reduction of the endothelial dysfunction and SMC proliferation, inhibition of macrophages migration and adhesion [165], decrease of proinflammatory cytokines and oxidative stress [166], and stabilization of atherosclerotic plaque [165].

#### 4.3.4. Pagln

A novel gut microbiota metabolite, phenylacetylglutamine (PAGln), was associated with cardiovascular disease and major adverse cardiovascular events in an independent cohort. Gut microbiota facilitates the conversion of phenylalanine into phenylacetic acid, with subsequent host generation of PAGln in humans and phenylacetylglycine (PAGly) in rodents, which mediates platelet activation and thrombosis through G-protein coupled receptors [167]. 

### 4.4. Oxidative Stress Biomarkers

Microscopic studies have documented the differences in the local inflammatory response for areas with vulnerable atheroma plaques compared to areas with stable atherosclerotic plaques. This difference in the local immune response has been correlated with mitochondrial dysfunction of ECs, which will lead to changes in local metabolism and cellular respiration, these cells being the target of oxidative stress [168]. In addition to the classical role of energy production and regulation of cellular metabolism, mitochondria are also responsible for the signaling function, the balance of cellular ions and the generation of oxygen free radicals and reactive oxygen species (ROS), such as superoxide anion (O^·−^), hydroxyl radical (OH^·^), and hydrogen peroxide (H_2_O_2_) as well as reactive nitrogen species (RNS): RNS-nitroxyl anion (NO−), nitrosonium cation (NO+), nitrate ((NO_3_)^·−^), and S-nitrosothyols (RSNO) [169]. Even if reactive species are produced almost continuously during aerobic cellular metabolism, their level is low, and in this small amount, they participate in various physiological processes, such as phagocytosis, immune reactions, or oxidative phosphorylation, and the antioxidant system can control the level of reactive oxygen species by neutralizing them. Cellular degradation occurs if the antioxidant system is no longer able to control the level of ROS and an increased level of ROS will determine genetic mutations in DNA-mitochondrial cells and cause the destruction of proteins and other cellular molecules [170].

Thus, oxidative stress occurs when there is an imbalance between the production and neutralization of oxygen free radicals, and the immediate consequence is the impairment of mitochondrial respiratory chain enzymes with the transition from aerobic to anaerobic metabolism and augmented production of oxygen free radicals. Furthermore, a vicious circle closes that leads to cellular and tissue destruction with the formation of a proinflammatory environment, and classical cardiovascular risk factors, such as smoking, dyslipidemia, hypertension, and diabetes accelerate this process [171]. 

Alleviating oxidative stress and inflammation is essential in the treatment of atherosclerosis. The classes of drugs with an antioxidant and antiinflammatory role according to the data published in the literature are statins, fibrates, oral hypoglycemic agents from the sulfonylureas and glitazone class, and Coenzyme Q 10 [172,173,174]. Mitochondrial dysfunction seems to be a key player in the proinflammatory response and chronic inflammation of the arterial wall, which eventually leads to the progression of atherosclerosis. Other biomarkers of endothelial dysfunction caused by mitochondrial dysfunction and oxidative stress are metabolic biomarkers.

### 4.5. Metabolic Biomarkers 

Plasma amino acids are new metabolic biomarkers able to identify patients at risk of developing a major cardiovascular event because endothelial dysfunction observed in the early stages of the atherosclerotic process is characterized by different levels of amino acids compared to the healthy population. Analyzing the plasma of a patient with endothelial dysfunction, experimental studies have shown that the increased level of L-Arginine and the low level of citrulline, these two amino acids being correlated with defective production of nitric oxide. Nitric oxide is a special signaling molecule formed by the action of the enzyme nitric oxide synthase on L-arginine. In response to this reaction, citrulline and Nitric Oxide are formed [175,176]

An important aspect of the atherosclerosis process is that nitric oxide synthase is present in three isoforms: eNOS, iNOS, and nNOS. Under physiological conditions, nitric oxide formed by the action of eNOS has a cardiovascular protective role, antiinflammatory, antiplatelet, inhibitor of migration and adhesion of inflammatory cells, and antioxidant by maintaining an increased amount of Glutathione necessary for the inhibition of reactive oxygen species [175]. Nitric oxide maintains an optimal level of glutathione with an antioxidant role by inhibiting the enzyme PKM2 (pyruvate-kinase M2), the glycolytic enzyme involved in glucose metabolism with pyruvate formation, a source for ATP, which allows the metabolism of glucose-6-phosphate on the pentose phosphate pathway with increased generation of NADPH and Glutathione. 

In pathological conditions, such as increased inflammatory status with cytokine release corresponding to the process of atherosclerosis, the iNOS (nitric oxide-nitric synthase inducible) isoenzyme produces nitric oxide in an increased quantity that will promote the inflammatory state and will aggravate the lesions at the level of the vascular endothelium. Nitric oxide causes cellular damage both on its own and through its ability to interact with the superoxide anion (O_2_^−^) and the formation of peroxynitrite (ONOO^−^), a reactive oxygen species with increased destructive activity [171]. Thus, an optimal level of nitric oxide at the endothelial level is necessary to maintain the metabolic balance of endothelial cells and protection against oxidative stress; any change in its synthesis is able to contribute substantially to the augmentation of the atherosclerosis process, an aspect that can be identified early by analyzing the markers of abnormal synthesis: L-arginine and citrulline [176].

Another interesting aspect observed in patients with endothelial dysfunction is the increased levels of cysteine (Cys) and Cistationine (Cth). These two amino acids are closely related to the synthesis of H2S (hydrogen sulfides), which is a new signaling molecule, part of the gas transmission family together with NO and CO and identified as having a cardiovascular protective role. It has been shown that H2S can inhibit multiple ways of development of atheroma plaque, such as limiting the formation of ox-LDL, slowing down leukocyte adhesiveness, formation of foam cells, and the proliferation of smooth muscle cells and endothelial cells, thus diminishing the local inflammation, which prevents the process of atherosclerosis. The way H2S exerts its cardiovascular protection effect is by participating in the S-Sulfhydration process, a process involved in post-translational changes that ensure the much faster exercise of the activity of over 3400 proteins [177]. 

The total level of H2S comes from both exogenous and endogenous sources through the activity of three important enzymes, CSE (cystathionine γ-lyase), CSB (Cystathionine-β-synthase), and 3-MST (3-mercaptopyruvate sulfurtransferase), and at the endothelial level, CSE is the enzyme responsible mostly for the synthesis of an optimal concentration of H2S. This acts on the Cth substrate to form H2S and Cys. In the plasma of patients with vascular dysfunction, abnormally high levels of these two amino acids are identified; thus, it becomes clear that the enzymatic activity is reduced with low production of H2S and loss of cardiovascular protective effect. Recent studies that have focused on the comparative analysis of plasma Cth levels in two groups, one consisting of patients with evidence of atherosclerotic plaque and one consisting of patients without atherosclerotic lesions; it has been shown that the increased level of Cth is closely correlated with the development of atheroma plaque. This produces a new molecule that can be used as a metabolic biomarker to identify patients with endothelial dysfunction who are at high risk for cardiovascular events [178].

Moreover, by the evidence brought in favor of H2S as an inhibitor of the atherosclerotic process, a new possible pharmaceutical target for cardiovascular prevention is identified: The CSE enzyme. It was observed that the more intense the local degree of inflammation, the lower the enzymatic activity and the level of H2S becomes inadequate, all leading to the promotion of the process of atherosclerosis. When the optimal level of H2S that is maintained under physiological conditions by the CSE enzyme was obtained, it was observed the restoration of vascular mechanics in response to the flow and the activity of B-integrin ensures a balance of the endothelial cell [179]. 

This result was recently transposed to humans using 1000 mg of methylsulfonylmethane (MSM) with donor polysulfide function in a group of six patients with endothelial dysfunction in whom recovery of vaso-mechanical activity was observed compared to the placebo-treated control group [180]. 

## 5. Proteomics of Vulnerable Plaques

Proteomic techniques achieve a profile of proteins, biomarkers that have high sensitivity and specificity for the identification of unstable atherosclerotic lesions. Thereby, the role of proteomics is to identify the local proteins involved in the atherosclerotic process and to evaluate the body’s response to this vascular aggression by analyzing the circulating proteins [2].

Proteomic studies use advanced techniques to separate the proteins by multidimensional liquid chromatography and to identify them by mass spectrometry, and thus, it allows the analysis of thousands of proteins simultaneously and from different sources, tissues, or plasma. In addition, it allows the comparison of different cells under normal or pathological conditions [181].

In the future, proteomics will have an important role in the primary and secondary prevention of atherosclerosis, because it allows a more thorough explanation of the pathophysiological processes that are involved in atherosclerosis and the identification of new biomarkers with a role in the progression and rupture of the atheroma plaques that can be therapeutic targets [2]. 

Applying proteomic methods to tissue samples obtained from atherosclerotic lesions from men and women can be explained the difference in cardiovascular risk between them. Previous clinical studies have shown that there are significant differences between the sexes when it comes to atherosclerotic development, with women before menopause showing protection against acute cardiovascular events compared to men [182]. Histological examinations have shown that women in the premenopausal phase develop a relatively stable atheroma plaque, with a reduced risk of rupture, while postmenopausal women have plaques with much more hemorrhage and a thinner fibrous cover. The hormonal distribution cannot fully explain these morphological differences, since the most significant hormonal imbalance is the level of estrogen, which has a vasodilator role by stimulating the secretion of nitric oxide. Even though there are also some antioxidant properties of estrogen, an explanation of this difference in the clinical and morphological characteristics according to gender should also be sought at the molecular level [182].

**Figure 3:**
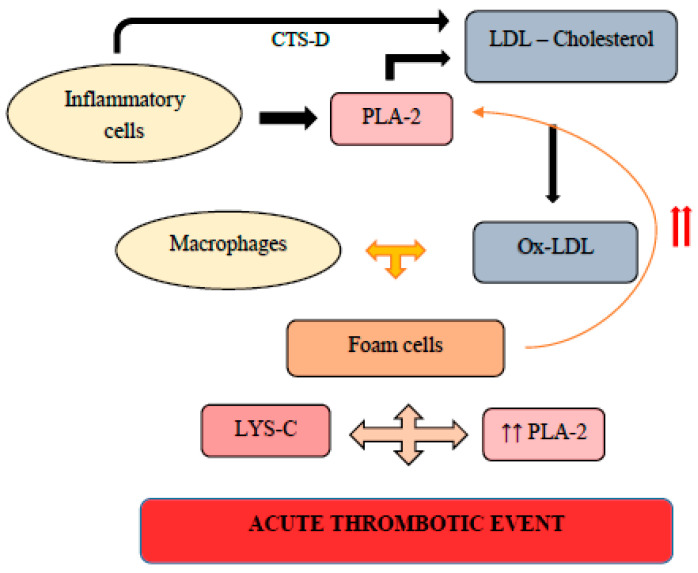
Inflammatory proteins involved in atherosclerotic plaque instability detected in tissue samples. CTS-D, Cathepsin-D; LYS-C, Lysozyme-C; ox-LDL, oxidized-LDL; PLA-2, Phospholipase-A2.

Proteomic studies applied on samples of atherosclerotic plaques obtained from groups of men and women with similar clinical characteristics have shown that there is a greater number of inflammatory proteins in atherosclerotic plaques from men, in particular, phospholipase A2 and lysozyme C. As can be seen in Figure 3, inflammatory cells in the arteries involved in the atherosclerotic process secrete Phospholipase-A2 (PLA-2) as a response to the aggression of various factors on the endothelial wall. This enzyme acts on LDL cholesterol to form oxidized LDL. Macrophages in the subendothelial space show an increased affinity for oxidized LDL in the immune system’s attempt to eliminate toxic metabolic products. In this process, macrophages turn into foam cells that secrete an even greater amount of PLA-A vicious circle is closed and this augmentation of the secretion of PLA-2 leads to the next step—the amplification of the inflammatory process capable of destabilizing the atherosclerotic plaque [182].

Lysozyme C (LYS-C), an enzyme released from subendothelial macrophages, is also involved in the rupture of the atheroma plaque and in acute cardiovascular events. Starting from this pathophysiological mechanism, the classical therapies that use statins aim to lower the level of LDL cholesterol, the reasoning being that if the substrate is reduced, the inflammatory process is slowed down [182]. By using proteomics, new therapeutic target scan be discovered. Since PLA-2 and LYS-C are enzymes present in the early stages of the atherosclerotic process and play an important role in acute cardiovascular events, they not only represent markers of instability, but can also be therapeutic targets to reduce overall mortality. Thus, clinical studies using Varespladib (a non-specific pan-sPLA2 inhibitor) have been conducted to reduce PLA-2 and LYS-C to prevent the rupture of the atherosclerotic plaques [182,183]. Further studies are needed to introduce this therapy into clinical practice.

Another interesting feature observed in the analyzed samples obtained from women showed that the level of the acute-phase proteins that contribute to the stabilization of atheroma plaque, such as alpha-1-acid glycoprotein 2 and alpha-1-anti chymotrypsin, is higher compared to the level of those present in samples collected from men. More than that, another difference is the amount of serine protease inhibitors that play a major role in the coagulation cascade (serpin C1, serpin D1). Both serpin C1 and serpin-D1, known as antithrombin-III and heparin-cofactor 2, respectively, inhibit the formation of thrombin, with a major role in the process of blood clotting and thrombus formation, a process favored by the rupture of the atherosclerotic plaque. These proteins are more abundant in women compared to men, which supports the hypothesis that the different proteomic profile provides a different level of protection for the benefit of the woman. Both the -C1 and the -D1 serpine have, in addition to sex-related variations, an age-dependent variation. This could be an explanation for the different risk percentages seen in younger women compared to older women [182].

Another group of proteins identified by proteomic methods is transport-related proteins. Afamin is a vitamin E-binding glycoprotein that shows a negative correlation with the inflammatory markers CRP and IL-It was also described to have a positive correlation with the value of HDL cholesterol, and its low level is a risk factor in the development of metabolic syndrome, increasing the risk for cardiovascular diseases. Proteomics studies have shown an abundance of afamin and zinc-alpha-2-glycoprotein in samples obtained from women, both proteins showing antiinflammatory properties and playing a role in the stability of the atheroma plaque [182]. 

In the progression and rupture of the atheroma plaque, there are several pathophysiological processes (inflammatory, oxidative stress, thrombotic) that present cascade activation and self-rejuvenation. As a result, the multiple-markers strategy was developed for the evaluation of atherosclerotic plaques at risk of rupture and clinical instability. Proteomics analyze at a microscopic level the various components that are structurally and functionally related in atherosclerosis development. Thus, by using proteomic methods applied to samples obtained after performing endarterectomy in symptomatic and asymptomatic patients, several proteins (Cathepsin-D, Matrix Metalloproteinase-9, S1001A8, S100A9, and galectin-3 binding proteins) have been identified to play a role in stratifying the risk of rupture of plaque [184].

Cathepsin-D is an enzyme detected by proteomic techniques consisting of high purity monocyte isolations and analysis using 2D electrophoresis and mass spectrometry. The enzyme is found not only in circulating monocytes but also in macrophages, representing one of the markers for differentiating monocytes into macrophages [184]. Cathepsin-D is the enzyme with the most important proteolytic action at the lysosomal level and has the role of degrading intracellular proteins and those coming from endocytosis [185]. In blood vessels with atherosclerotic degeneration, this enzyme is secreted in a significant amount into the extracellular space by macrophages and performs hydrolytic changes in LDL cholesterol and, ultimately, favors the formation of foaming cells, precursors of atherosclerotic plaque. As can be seen in Figure 2, this enzyme has a key role in atherosclerosis along with PLA-2 and many other enzymes. In addition, Cathepsin-D is not only a promoter of atherosclerosis, but also an enzyme involved in the instability of the atheroma plaque by generating apoptosis of macrophages and foam cells. The instability of plaque is the crucial step of acute vascular events, and prevention therapies try to delay this phase as much as possible. Overexpression of this enzyme has been noticed in patients with the acute coronary syndrome, as opposed to the lower levels found in healthy subjects, and the proenzyme-proform-D appears to be higher in healthy subjects. These results led to the analysis of the plasma level of the mature soluble form of Cathepsin -D, which appears to increase in patients with vulnerable plaques and even higher in those with acute thrombotic events. Several studies have shown that there is an association between the level of Cathepsin-D and cardiovascular events by initiating and maintaining proteolytic processes that lead to instability and rupture of the atherosclerotic plaque. Thus, complementary to traditional therapies, medication with a role in lowering the level of Cathepsin-D, could improve the prognosis of patients in both primary and secondary prevention of atherosclerosis [186]. Moreover, some studies have shown that patients undergoing statin treatment had lower levels of Cathepsin-D compared to patients who did not undergo any treatment [186]. Studies conducted to determine the role of Cathepsin-D have shown that, in addition to the high level of Cathepsin-D found in the cells and plasma of cardiovascular patients, other members of his family also appear to be involved in the total risk of thrombotic events. In patients who have experienced at least one cardiovascular event, the level of cathepsin L and its inhibitor, Cystatin-B, is higher compared to the control group [187]. Cathepsin-L plays the same role as homologous cathepsin-D, but more in-depth studies have shown that Cathepsin-L, in addition to involvement in plaque degradation, is also responsible for the process of remodeling of the endothelial wall in response to stress induced by turbulent flow. Thus, cathepsin-L acts even earlier in the pathophysiological mechanism of atherosclerosis. Studies have also shown that cathepsins have a higher serum and tissue level in smoking and diabetic patients [185,186,188] 

Matrix metalloproteinase-9 (MMP-9), known as gelatinase B, is a member of a family, along with 28 other enzymes with a proven role in plaque instability due to its involvement in the remodeling of the extracellular matrix. Studies have reported several cell types as a source of MMP-9: macrophages, SMCs, and ECs [184]. The secretion of MMP-9 from ECs is stimulated by shear stress that acts on the vascular walls. MMP-9 degrades the extracellular matrix and causes inflammatory cells to infiltrate the subendothelial space that will secrete an even greater amount of MMP-MMP-9 in the atherosclerotic plaque promotes the inflammatory reaction by stimulating the chemotaxis of immune cells and maintaining inflammation. Thus, MMP-9 stimulates the migration of VSMC, which increases the secretion of VEGF (vascular endothelial growth factor), favoring neovascularization, a process that indirectly affects the stability of the plaque [94]. MMP-9 can be identified both in tissue and serum level with pathophysiological implications in different stages of cardiovascular diseases. The increased level of tissue MMP-9 is associated with the instability of the atherosclerotic plaque, and the increase in serum levels is the marker of acute cardiovascular events, such as NSTEMI or STEMI, due to secretions of MMP-9 by the ruptured plaque [189]. When the level of MMP-9 is low, the proliferation rate of monocytes and macrophages is reduced and the evolution of atherosclerosis is slowed down. Studies have shown that patients with an increased level of MMP-9 also experience increased levels of C-reactive protein and instability of the atherosclerotic plaque by increasing inflammation. When comparing the temporal evolution of the level of MMP-9, it is shown that high levels of circulating enzyme are recorded in early cardiovascular events, even earlier than high-sensitivity troponin and returning to baseline after a few days, along with the restoration of the arterial wall. This interesting fact can place MMP-9 among the other screening biomarkers, since its level is increased in the early stages of plaque instability. Acting at this very early stage of the coronary event can allow us to save the heart muscle and minimize the damage caused by the formation of thrombus and arterial occlusion [190]. 

Galectin-3 is an inflammatory mediator that promotes atherosclerosis by affecting multiple key cells with a structural and functional role in the arterial wall, such as ECs, SMCs, and immune system cells. Studies showed that the level of Galectin-3 increases in unstable plaques compared with stable regions of atherosclerotic areas from the same patient. Galectin-3 is secreted as monomers, but when the number of monomers is high, Galectin-3 monomers bind to form pentamers that accelerate the injury on the vascular wall. Multiple cells can secrete Galectin-3, including macrophages, monocytes, neutrophils, and structural cells from various tissues [184,191]. The initial step in the atherosclerotic process is the injury of the ECs by the ox-LDL and the presence of Galectin-3 aggravates the lesions. Once the ECs are dysfunctional, inflammatory molecules released in the process attract the monocytes with further differentiation into macrophages and Galectin-3 accelerates this process. Despite the monocytes’ expression of Galectin-3 on their surface, the main source of this marker is represented by the macrophages and foam cells. In tissue samples, Galectin-3 accumulates in areas with a high number of macrophages and foam cells and increases the uptake of the ox-LDL by the macrophages. By disturbing lipid metabolism and promoting the inflammatory process, Galectin-3 is a marker for plaque extension and instability. Even if the SMCs do not represent the main source for Galectin-3, the proliferation process of SMCs is increased in presence of Galectin-3 [191]. Acting in this multimodal way to increase the atherosclerotic process, Galectin-3 has a central place and represents a target for prevention therapies. At present, statins have shown their effect on lowering the level of Galectin-3 in various studies [192], but other therapies, such as Quinapril, mineralocorticoid receptor antagonists, and N-acetyllactosamin, were shown to have a positive effect on the level of Galectin-3 [193,194,195]. On the other hand, Doxazosin exacerbates the expression of Galectin-3 in cardiomyocytes [196]. These results may offer new perspectives on atherosclerosis prevention by using Galectin-3 inhibitors. 

In the pathophysiological process of acute thrombotic events, platelets play a significant role, and antiplatelet therapy is an important pharmacological method in the primary and secondary prevention of acute coronary syndromes. The rupture of the atherosclerotic plaque favors the adhesion of platelets to the extracellular matrix of the vascular wall and promotes the formation of a thrombus and the secretion of specific proteins by platelets. Since platelets are cells without a nucleus, genetic studies cannot be conducted on this pathophysiological mechanism. Moreover, platelets are cells with an increased capacity for secretion. Thus, proteomics allowed us to identify more than 300 proteins secreted in the thrombotic phase of the atherosclerotic process, most of them being absent in the serum of healthy people. Moreover, platelets secrete many inflammatory mediators and growth factors from their storage granules that stimulate inflammation and proliferation of smooth muscle cells. Proteins such as secretogranin III, cyclophilin A, calumenin, P-selectin, and TNF-α appear to be important promoters of inflammation and thrombosis and therapeutic targets in the future that can allow us to decrease the frequent hemorrhagic risk of the classical antiplatelet therapy. Several studies have applied proteomic technology to platelet samples to identify new biomarkers of acute thrombotic events [181,197]. 

The proteins thus identified can be classified into different functional groups that have different roles in favoring the instability of the atheroma plaque: proteins responsible for platelet activation, signaling proteins, cytoskeletal proteins, proteins involved in the metabolic process, and proteins with an antioxidant role (Table 3) [181]. A system with therapeutic potential is the antioxidant protein system, the use of antioxidant drugs having a role in stabilizing the atheroma plaque [181].

A summary of studies regarding the circulating biomarkers used in the diagnosis of vulnerable atherosclerotic plaque vs. non-vulnerable plaque is found in Table 4. 

### Strengths and Limitations of the Review

This review summarizes the current data from the specialty literature regarding biomarkers that play a significant role in the pathogenesis of vulnerable atherosclerotic plaque. Our work is meant to help clinicians working in cardiovascular medicine, express new targeted approaches in terms of pathophysiology, diagnosis, risk stratification, and therapeutic tools, in an effort to reduce the morbidity and mortality of patients at risk of developing acute cardiovascular events.

Several biomarkers have been studied in only few, limited scientific papers, underlying the need for continuous and rigorous further research. Hence, the lack of metanalysis focusing on their prognostic value as a multimarker strategy in patients at risk of developing acute cardiovascular events encompasses our study limitation.

## 6. Conclusions

Stratifying the patients’ risk of developing acute cardiovascular events is very important, having prognostic and therapeutic consequences.

The formation of vulnerable atherosclerotic plaque that also determines the notion of a vulnerable patient at risk of cardiovascular events involves multiple pathophysiological processes: inflammation, oxidative stress, thrombogenesis, and metabolic and epigenetic changes. For risk stratification, the multivariate approach is used by identifying biomarkers involved in different pathophysiological stages, thereby increasing the sensibility and specificity of the prediction of a major cardiovascular event. New genomics and proteomics studies offer the possibility to identify new methods of stratification of patients and new potential therapeutic targets.

Among the inflammatory biomarkers with a prognostic role in vascular events, we mention CRP, IL-6, and MMP-Metabolic markers that can predict the risk for major vascular events are: increasing the serum value of L arginine, decreasing citrulline level, and increasing cystathionine and cysteine. The change in the intestinal microbiota identified by the increase of TMAO and LPS and the decrease of SCFA predisposes the patients to high cardiovascular risk in the long term.

Epigenetic biomarkers have a great potential for identification vulnerable plaques. miR-15a-5p, miR-30e-5p, miR-92a-3p, miR-199a-3p, miR-221-3p, and miR-222-3p were associated with coronary plaque necrotic core volume, while miR-122-5p and miR-223-3p were significantly upregulated both in plaque tissue evaluation and in serum in patients with unstable coronary syndromes. Clearly, larger studies are needed to prove their usefulness in clinical practice. 

By using proteomics methods, certain proteins, when modified together (Cathepsin-D, MMP-9, and galectin-3 binding proteins), have been identified to have a role in stratifying the risk of atherosclerotic plaque rupture. 

## Figures and Tables

**Table 1:** Non-invasive imaging of vulnerable plaque classified by the mechanisms of vulnerability.

Mechanism of Plaque Vulnerability	CTCA	PET Techniques
Inflammatory cell infiltration (macrophage activation)	Fat attenuation index (FAI) CTCA Radiomics [36] Radiotranscriptomics	18F-fluorodeoxyglucose (18F-FDG)
		68Ga-somatostatin receptor subtype 2 (68Ga-DOTATATE) [37]
		11C-translocator protein (11C-PK11195)
		18F-fluorocholine (18F–FCH)
	FRP CTCA	89Zr-DFO-Gal3-F(ab’)2 mAb [38]
		In-DOTA-JR11PET/CT [39]
Neo-angiogenesis		18F-glycoprotein IIb/IIIa platelet receptor (18F-GP1)
Hypoxia		18F-fluoromisonidazole (18F-FMISO)
		18F-HX4
Apoptosis	‘Napkin-ring sign’ on CCTA	18F-annexin *V*
Calcification and microcalcification	Spotty calcification on CCTA	18F-NaF and 18F-FDG hybrid PET and MR [40]
Hemodynamics and shear stress	CFD CCTA	18F-NaF PET [40]
Intraplaque hemorrhage	FLECT NIR-AF [41]	18F-NaF PET [40]
CCTA, coronary computed tomography angiography; CFD, computational flow dynamics; FLECT, fluorescence emission computed tomography; NIR-AF, Near infrared auto-fluorescence; PET, positron emission tomography; 11c, carbon-11; 68Ga, gallium-68; 18F, fluorine-18; 18F-NaF, 18F-sodium fluoride; 124i, iodine-124.		

**Table 3:** Protein biomarkers classified into different functional groups by their role in favoring the instability of the atheroma plaque.

PLATELET ACTIVATION	Upregulated with high affinity to collagenous structures in the core region of the atherosclerotic plaque
Glycoprotein VI receptor	
αIIbβ3 receptor	
SIGNAL PATHWAYS	Over-expressed—inducing cell growth, survival, adhesion, invasion, and migration
Integrin-linked kinase	
Sarcoma protooncogene tyrosine-protein kinase	
CYTOSKELETON-ASSOCIATED PROTEINS	Downregulated in human atherosclerotic plaques, which indicates the tendency to limit the platelet activity
Talin	
Vinculin	
A and β tubulins	
Vimentin	
F- actin capping	
ENERGY METABOLISM-RELATED PROTEINS	
Glyceraldehyde-3-phosphate dehydrogenase	
Pyruvate kinase	
Lactat dehydrogenase	
ANTIOXIDANT PROTEINS	Lower expression that facilitates platelet activation and allows advanced oxidation protein products to maintain chronic inflammation
Glutathione-S-transferase	
Manganese superoxidase dismutase	

**Table 4:** Clinical and pre-clinical studies regarding circulating biomarkers involved in the diagnosis of vulnerable atherosclerotic lesions.

Biomarker.	Study Conclusion	References
hs-CRP	Hs-CRP levels correlated with carotid plaque instability in dyslipidemic male patients.	Scimeca M. 2021 [117]
sCD40/CD40-L	SCD40 levels were correlated with the severity of carotid atherosclerosis and future cardiovascular events; intra-plaque levels of sCD40 were associated with vulnerable plaque properties.	Shami A. 2021 [87]
MMPs	MMP-1, MMP-3, and MMP-12 levels were positively associated with vulnerable plaque phenotype and the occurrence of major acute vascular events in patients with carotid atherosclerotic disease. Circulating MMP-9/NGAL complex levels and NGAL levels were associated with vulnerable atherosclerotic plaques in patients with carotid artery stenosis.	Hu w. 2017 [95] Eilenberg W. 2017 [98]
MPO	MPO activity was associated with features of vulnerable plaques in Apo E−/− mice. MPO was suggested to be a pharmacological target against atherosclerosis and a marker for non-invasive imaging of high-risk unstable plaques.	Rashid I. 2018 [105]
MCP-1	MCP-1 plaque levels were associated with histopathological, molecular, and clinical characteristics of vulnerable plaques in carotid endarterectomy samples.	Georgakis MK. 2021 [109]
C5b-9	C5b-9 correlated with the stability, severity, and outcome of carotid lesions in patients with acute ischemic stroke.	Si W. 2019. [113]
TMAO	In a tandem stenosis mouse model, TMAO levels were correlated with several features of plaque instability regarding inflammation, platelets activations, and intraplaque hemorrhage.	Koay YC. 2021 [160]
Cathepsin D	Cathepsin D serum concentrations were associated with the presence of coronary artery disease. Among other makers, cathepsin D was correlated with the high-risk profile of atherosclerotic plaque from endarterectomy samples.	Mohammadpour AH. 2018. [186] Langley SR. 2018 [184]

## Data Availability

Not applicable.
